# Endovascular Treatment of Intracranial Artery Dissection: Clinical and Angiographic Follow-Up

**DOI:** 10.1155/2013/968380

**Published:** 2013-07-22

**Authors:** Reza Mohammadian, Ali Akbar Taheraghdam, Ehsan Sharifipour, Reza Mansourizadeh, Ali Pashapour, Mohammad Shimia, Ghaffar Shokouhi, Moslem shakeri, Ali Hashemzadeh

**Affiliations:** ^1^Neuroscience Research Center, Tabriz University of Medical Sciences, P.O. Box 51665-348, Tabriz, Iran; ^2^Neurology and Neurosurgery Department, Alinasab Hospital, Tabriz, Iran; ^3^Neurosurgery Department, Tabriz University of Medical Sciences, Tabriz, Iran

## Abstract

*Background*. Intracranial artery dissections are rare and many controversies exist about treatment options. The aim of this study was to evaluate the efficacy and safety of the endovascular approach in patients with an intracranial dissection presenting with different symptoms. *Methods*. We prospectively evaluated the clinical features and treatment outcomes of 30 patients who had angiographically confirmed nontraumatic intracranial dissections over 4 years. Patients were followed up for 17 months, and their final outcomes were assessed by the modified Rankin Score (mRS) and angiography. *Results*. Sixteen (53.3%) patients had a dissection of the anterior circulation, whereas 14 (46.7%) had a posterior circulation dissection. Overall, 83.3% of the patients suffered a subarachnoid hemorrhage (SAH). Grade IV Hunt and Hess score was seen in 32% of the SAH presenting cases. Parent artery occlusion (PAO) with coil embolization was used in 70% of the cases. The prevalence of overall procedural complications was 23.3%, and all were completely resolved at the end of follow-up. No evidence of in-stent occlusion/stenosis or rebleeding was observed in our cases during follow-up. Angiography results improved more frequently in the PAO with coil embolization group (100%) than in the stent-only-treated group (88.9%) (*P* = 0.310) and the unruptured dissection group (5/5, 100%) in comparison with the group that presented with SAH (95.8%) (*P* = 0.833). *Conclusion*. Favorable outcomes were achieved following an endovascular approach for symptomatic ruptured or unruptured dissecting aneurysms. However, the long-term efficacy and durability of these procedures remain to be determined in a larger series.

## 1. Introduction 

Arterial dissections are delineated by sudden disruption of the endothelium, the intima, and the internal elastic lamina with subsequent influx of circulating blood into the media. The pathogenesis of most intracranial artery dissections has been debated, and their etiology involves both extrinsic and intrinsic factors as well as defective repair mechanisms. Some of the factors associated with arterial dissections include hypertension, smoking, inflammatory diseases, genetic predisposition, fibromuscular dysplasia, collagen disease, and trauma [1]. Symptoms in patients with intracranial dissection can be related either to the mass effect, ischemia, or subarachnoid hemorrhage (SAH), or, in rare cases, to a combination of different presenting symptoms [2, 3] that can be described by the Mizutani et al. [4] classification of dissection pathomechanisms. A dissection diagnosis is typically made by angiographic appearance such as a double lumen, focal irregularity of the vessel wall, preaneurysmal narrowing, and fusiform dilatation. Magnetic resonance imaging (MRI) reveals intramural thrombus, vessel wall irregularities, or flap. Treatment options remain controversial and depend on the clinical presentation (SAH or others). They include medical therapy, as well as endovascular and surgical interventions. The introduction of endovascular methods has added an attractive minimally invasive therapeutic approach and spares the patient some of the hazards associated with craniotomy and open surgery. Endovascular therapy can be basically deconstructive such as by using occlusion of main artery where the aneurysm is derived (parent artery occlusion (PAO)) with/without coil embolization or reconstructive such as stenting with/without coiling based on whether the parent artery must be preserved or not [5]. The aim of this study was to report our clinical and follow-up results in 30 patients who underwent endovascular treatment for posterior or anterior circulation dissections and presented with different symptoms.

## 2. Methods 

### 2.1. Patients

Patients with acute neurological symptoms suspected to be intracranial dissections and who were referred to our institute from May 2008 to June 2012 were evaluated prospectively. 

The diagnosis of an intracranial artery dissection was based on medical records and all neuroradiological images of the cases. Cases that fulfilled the following criteria were included: sudden onset of ischemic or hemorrhagic symptoms, cerebral angiography showing characteristic arterial dissection findings [6, 7], arterial dissection in the intracranial portion on cerebral angiography, and no obvious atherosclerotic changes found in other intracranial arteries on cerebral angiography. Finally, the cerebral angiography findings must have been consistent with the clinical symptoms. 

The cerebral angiography findings to confirm arterial dissection included (1) the double-lumen sign [8, 9], (2) stenosis with dilatation (the pearl and string sign) [10–12], (3) stenosis without dilatation (the string sign) or occlusion (tapered occlusion), (4) extensive stenosis but not segmental stenosis on initial angiography, (5) resolution of stenosis or occlusion seen on follow-up angiography [13, 14], and (6) dilatation without stenosis (discoloration of the affected artery around the aneurismal dilatation, which was considered to be due to intramural hematoma). Diagnostic four-vessel angiography was conducted in all patients, and a general anesthetic was administered if required. Angiograms were assessed for size, shape, and location of the dissecting aneurysm with respect to the major branches and collaterals (i.e., the presence or absence of the contralateral vertebral artery or posterior communicating arteries). Each lesion was examined for evidence of extension of the dissection into adjacent arterial segments, including the posterior inferior cerebellar artery (PICA) and basilar artery. 

The balloon test occlusion was performed at a site that best simulated the anticipated therapeutic occlusion, without entering the dissected segment, depending on the patient's neurologic condition and stability. 

Patients were systematically anticoagulated with intravenous heparin using a standard protocol during the test occlusion [15]. Neurological testing was performed throughout the procedure with the patient under monitored anesthesia. If the patient remained at their neurologic baseline and the follow-up angiogram demonstrated adequate collateral flow, the decision was made about endovascular technique (stent only or PAO with coil embolization). Patients who had incidental findings of an intracranial dissection in which their symptoms were not related to their dissected arteries were excluded. Two in-stent only candidate patients with unruptured dissections were excluded. Finally, a total of 30 patients with 30 dissected arteries were identified and included in this study. All cases had fusiform dissections. The locations of the dissections were as follows: vertebral artery (VA) (V4 segment) in 12 patients, posterior cerebral artery (PCA) (P1 segment) in five patients, internal carotid artery (ICA) (supracavernous segment) in five patients, MCA (M1 segment) in five patients, and the ACA (A2 segment) in three patients. Twenty-five of the 30 patients had ruptured dissections that presented with bleeding (SAH), and almost the entire group had sudden onset headache and some degree of lethargy as common symptoms. All scored ≤4 on admission according to the Hunt and Hess scale [16]. Various symptoms were observed in the unruptured dissection (ischemic) cohort, including cranial nerve III palsy, ophthalmoplegia, TIA, left side hemiparesis, cerebellar infarction symptoms, and dysarthria. Standard protocols were used to evaluate all patients. Demographic features, vascular risk factors, anatomical locations of the dissections, baseline modified Rankin Score (mRS), baseline and postprocedure National Institutes of Health Stroke Scale (NIHSS), presenting symptoms, treatment method, final mRS, final follow-up angiographic outcomes and complications were recorded by an expert neurologist and presented in Table 1. Postprocedure NIHSS was calculated in hospital 24 hours after the endovascular treatment. Vascular risk factors included hypertension (receiving medication for hypertension or blood pressure >140/90 mmHg on repeated measurements), diabetes mellitus (receiving medication for diabetes mellitus, fasting blood glucose level ≥126 mg/dL), dyslipidemia (receiving lipid-lowering agents or an overnight fasting cholesterol level >200 mg/dL and low-density lipoprotein >100 mg/dL), and current cigarette smoking (current smokers or those who quit smoking for <6 months). A neuroradiologist informed the patients about the benefits and potential risks of endovascular therapy. Patients who gave informed consent were transferred to the angiography room. All patients were required to have no contraindications for endovascular procedures (renal failure, coagulopathy, or contrast allergy). Patients were considered eligible for stent therapy only if they had no other therapeutic options and accepted the risk. The indications used for arterial dissection included vertebrobasilar insufficiency despite anticoagulant or antiplatelet therapy, contraindication to anticoagulants, contralateral vertebral occlusion or stenosis in a patient who was neurologically unstable or had clinical evidence of hemodynamic insufficiency, and documented poor collateral circulation. But some patients who underwent coil embolized PAO were qualified for stent implant but refused the procedure because of the risk and/or expense (because they could not afford the cost of treatment and were therefore refraining from doing it). A few patients wanted a stent but were not offered a stent implant due to technical difficulties (because of the anatomical location of the dissection or the lack of access to proper location). The angioplasty and endovascular techniques were performed by the same interventional neuroradiologist who implemented angiography via the transfemoral artery approach and under general anesthesia after obtaining informed consent. PAO with coil embolization was applied to 21 patients with an arterial dissection, and the stent-only method was used in the remaining nine. SAH was managed in the critical care unit including complete rest and avoidance of an adrenergic response. Patients received antispasmodic, antiepileptic, supportive treatment and vital sign monitoring. Dual antiplatelet therapy (80 mg aspirin daily and 75 mg clopidogrel) was given to the patients with unruptured acute intracranial dissections before and after procedures. Baseline activated clotting time was measured, and 12-hour anticoagulation therapy was started at the beginning of the procedure to maintain activated coagulation time >2-3 times the baseline value during the procedure, followed by a low amount of the drug for 24–48 hours after the procedure. Antiplatelet premedication was not administered to patients with a ruptured acute intracranial dissection, but a loading dose of dual antiplatelet therapy was given immediately after completion of the procedure. Dual antiplatelet therapy was extended for 3–6 months in both groups and was then changed to aspirin monotherapy for life. 

### 2.2. Techniques

The stent implant patients were administered local anesthesia and light neuroleptic anesthesia to allow continuous neurological monitoring. A 6F or 7F introducer sheath was placed in the right femoral artery. Full systemic heparinization was achieved by administering a 4000 IU bolus after placing a guiding catheter in the proximal region of the dissected artery followed by hourly boluses of 1000 IU to maintain an activated clotting time >250 seconds. Patients who presented with acute SAH were unable to undergo anticoagulation therapy. 

Stents were positioned across diseased segments so that they overlapped on each side of the dissected orifice. We used self-expandable stents to achieve appropriate luminal diameter and sufficiently narrow strut size to occlude the dissecting aneurysms. After a final angiogram check, the catheter was removed, and the sheath was left in the groin. The patient was moved to the neurosurgery intensive care unit for monitoring and received 1000 IU/hour heparin for the next 24 hours. Heparinization was discontinued 24 hours after treatment but was not reversed. Stent type was determined based on stent availability, operator preference, and the difference in vessel diameter between the proximal and distal portions of the affected segment. Technical success was defined by correct placement of the stent(s). PAOs were performed with Guglielmi detachable coils in 21 patients. All patients who underwent PAO and coil embolization had computed tomography (CT) scans 48 hours, 3 months, and 1 year after treatment. 

### 2.3. Follow-Up

All cases were followed up clinically through outpatient visits at 1, 3, 6, and 12 months and yearly thereafter until termination of follow-up (this will occur when the last patient has been followed up for 6 months). Patients were informed to visit their physicians immediately whenever neurological symptoms occurred. All patients were also advised to undergo a magnetic resonance angiography study 1 week after the procedure and then at each follow-up visit. The angiographic follow-up was performed 24 hours after the procedure then yearly or at the final follow-up visit along with digital subtraction angiography (DSA). The final follow-up angiography and DSA results were categorized into four classes: (A) complete obliteration (if the dissection sac was completely obviated or the parent vessel completely embolized), (B) partial obliteration (if the dissection sac clearly decreased in size but remained or the parent vessel was partially embolized), (C) stable (if the dissection sac revealed no progressive remarkable change in size and shape), and (D) exacerbated (if the dissection sac increased in size). The (A) and (B) categories were considered improved results, and the (C) and (D) categories were considered unimproved results. In-stent occlusion/stenosis and patency of the branch vessel or the perforators covered by the stents were also evaluated on the follow-up angiography. Each patient's clinical status at the last clinical follow-up was assessed by the final mRS. Safety of the procedures was evaluated by the incidence of any procedural-related complications, including adverse events during the procedure or within 30 days after the procedure. We evaluated the follow-up angiographic results according to presenting symptoms (SAH or non-SAH) and endovascular procedure type. 

### 2.4. Statistics

We compared the angiographic follow-up results (improved or unimproved) in the 29 intracranial arterial dissection cases (one patient died 10 days after the procedure) between the ruptured (*n* = 24) and unruptured (*n* = 5) groups and between cases who underwent stent only or PAO and coil embolization using the two tailed test. SPSS version 13.0 for Windows statistical software (Chicago, IL, USA) was used. All *P* values were two tailed, and a *P* ≤ 0.05 was considered significant.

## 3. Results 

**Figure 1:**
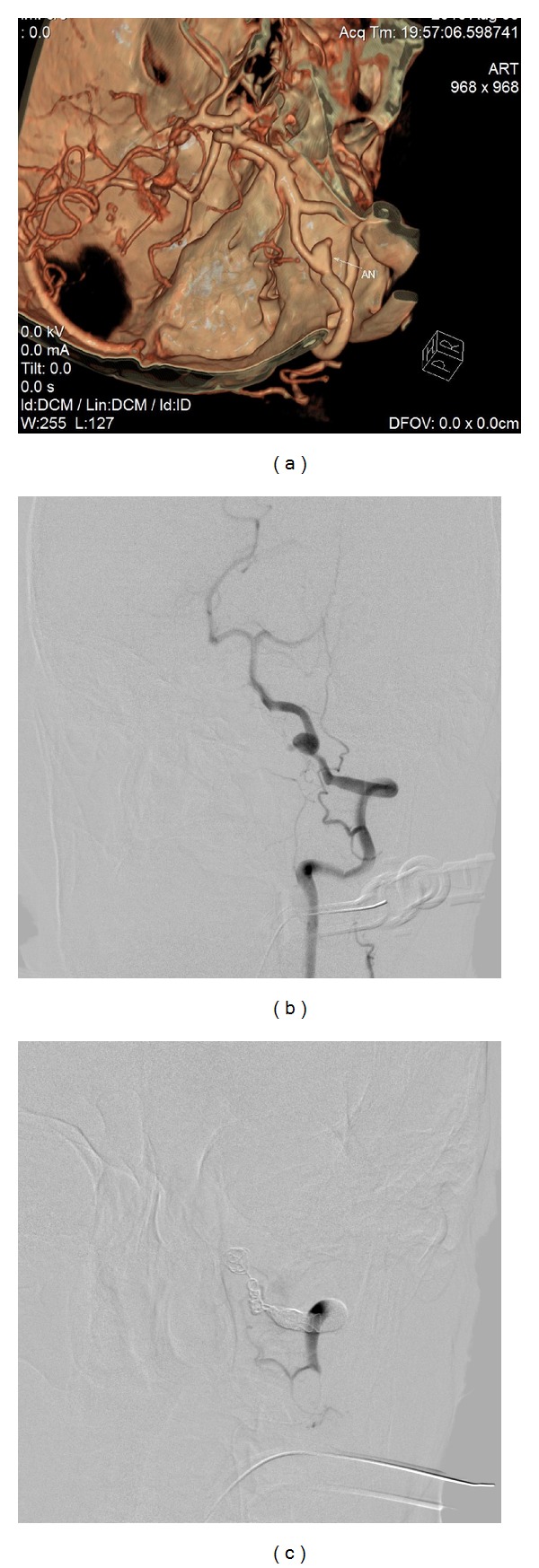
A 56-year-old hypertensive and smoker male patient presented with acute onset headache and loss of consciousness. Brain CT angiography revealed dissecting aneurysm in the distal segment of left vertebral artery. (a) This finding was confirmed by DSA. (b) The patient underwent coil embolization and parent artery occlusion. (c) Unfortunately, the patient died because of aspiration pneumonia 10 days later.

Overall, 30 patients were evaluated. Their clinical characteristics and angiographic imaging and clinical and follow-up results are summarized in Table 1. The prevalence of vascular risk factors in these cases was 73.3%, 43.3%, 33.3%, and 16.7% for hypertension, diabetes mellitus, cigarette smoking, and dyslipidemia, respectively. SAH as a presenting symptom was in 25 of 30 (83.3%) patients, and the dissection sites were VA (40%), ICA (16%), MCA (16%), PCA (13.3%), and ACA (13.3%). In patients who presented with SAH, the predominant site for lesions was the VA in 10 of 12 patients (83.3%) and the predominant angiographic finding was stenosis with dilatation in 20 of 25 patients (80%), while in patients with symptoms other than SAH was stenosis without dilatation in three of five patients (60%). A fusiform aneurysm that engaged VA/V4 was revealed in 12 cases and PCA/p1 in five. Of them, 14 cases presented with SAH, one with third nerve palsy due to the mass effect (case 15), and the remaining cases presented with cerebellar infarction (cases 10 and 16). Poor clinical grade of the bleeding cases (SAH) on admission, which was defined as grade IV on the Hunt and Hess score, was observed in eight of 25 patients (32%). Grade III was found in 60% and grade II in 8% of patients. Of the 25 ruptured arterial dissection cases that presented with SAH, four were treated with a single stent and the remaining 21 were treated by PAO with coil embolization. The five patients who presented with symptoms other than SAH were treated with a stent-only procedure, except for one patient (case 1) with a grade IV Hunt and Hess score who died due to aspiration pneumonia 10 days after PAO with embolization (Figure 1). All other patients completed the clinical follow-up at a mean of 17 months (range, 6–33 months), and we did not lose any cases to follow-up. 

The 21 patients who presented with SAH had excellent outcomes (final mRS scores: 0-1). The two remaining SAH cases did not have good final functional outcome because of their initial damage (cases 3 and 24; final mRS scores: 3 and 3). All five patients with symptoms other than SAH had excellent outcomes without any neurological deficits (cases 10, 15, 16, 21, and 28; all final mRS: 0). No procedural failures were observed during endovascular treatment. The prevalence of overall procedural complications was 23.3% (seven of 30 cases). The stent procedural-related complications included two cases with MCA dissection with new onset symptoms of putamen emboli (cases 24 and 25) on a CT scan performed 48 hours after the procedure (22.2%; two of nine case). Three cases with VA/V4 dissection with PICA involvement that were managed with PAO and coil embolization developed new onset symptoms of distal branch emboli of the PICA (cases 2, 3, and 8), and a patient with a PCA dissection developed new onset symptoms of thalamus emboli on a CT scan performed 48–72 hours after the procedure (19%; four of 21 cases) (Table 1). All these ischemic signs improved without aggravation in all patients, which resulted in good recovery according to the final mRS scores. 

No recurrent hemorrhage occurred during the mean follow-up period of 17 months in patients with SAH. No evidence of in-stent occlusion/stenosis was observed at the early and final follow-up angiograms of the stent-only managed cases, and branch vessel or perforator patency covered by the stents was good. No evidence of retrograde filling or leptomeningeal collateral supply of the vessel or recanalization of the embolized dissection was observed in cases treated by PAO. The final follow-up angiography and concomitant DSA study revealed that all cases showed improved results (A class) in patients who had complete obliteration of the dissection sac except for one stent-treated patient (case 25) who had an unimproved result. Improved angiography and DSA imaging results were more frequently observed in the PAO with coil embolization group (20/20, 100%) than those in the stent-only-treated group (8/9, 88.9%) (*P* = 0.310) and in the unruptured dissection group (5/5, 100%) compared with the group presenting with SAH (23/24, 95.8%) (*P* = 0.833), but neither of these was significantly different between cohorts (Table 2). 

## 4. Discussion 

Only a few large intracranial arterial dissection case series have been reported, but with advances in noninvasive angiographic diagnostic procedures it is being increasingly recognized [17]. The purpose of this study was to evaluate the periprocedural and follow-up outcomes of patients who underwent PAO with coil embolization or stenting as endovascular approaches for treating intracranial fusiform dissected aneurysms in the anterior or posterior circulation.

The endovascular approach to dissection of the intracranial artery can be divided into deconstructive (involving occlusion or sacrifice of the parent artery) and reconstructive (preserving blood flow through the parent vessel) procedures. Deconstructive endovascular techniques include proximal PAO with detachable coils and/or balloons and occlusion of the dissected segment of the vessel with coils and/or balloons. However, patients who cannot tolerate parent vessel occlusion or cannot be monitored for vessel sacrifice because of their poor underlying neurological condition secondary to the initial hemorrhage remain challenging cases. Reconstructive endovascular techniques consist of stent placement, including stent implant and stent-assisted coil embolization. These techniques preserve the parent vessel, which eliminates the need for revascularization when angiography demonstrates inadequate collateral flow or when the dissected segment involves major branch vessels [5, 18]. Coil embolization to directly occlude the affected artery segment has been suggested as an appropriate therapy for VA dissecting aneurysms [19, 20]. A study by Santos-Franco et al. on dissecting vertebrobasilar system aneurysms demonstrated that the stent-only technique is a promising approach, allowing for occlusion of the dissected aneurysm while preserving vessel patency and reconstructing the affected segment [21].

In an investigation by Sedat et al. on five patients with a PICA dissection that mainly presented with SAH, they applied the embolization (endovascular sacrifice of the PICA) technique, and their angiographic and clinical follow-ups were >3 years. They concluded that endovascular treatment is safe and effective in patients presenting with SAH [22]. Yoon et al., in their research on V4 segment VA dissections, revealed that endovascular treatments such as stent-assisted angioplasty or coil occlusion at the dissection site can be performed in selected patients with posterior fossa ischemic symptoms [23]. In our study, we revealed that stenting or PAO with coil embolization not only can be used safely for ischemic cases but also for hemorrhagic cases that have VA/V4 involvement. The final mRS score in our series was 0-1. Ruptured VA dissecting aneurysms presenting with a poor/good SAH grade can be managed safely with coil occlusion of the lesion and/or parent artery [24]. Albuquerque et al. conducted an investigation of 23 cases to evaluate treatment efficacy and outcomes of endovascular management of intracranial VA dissecting aneurysms. Twelve patients presented with poor-grade SAH. Treatments included coil occlusion of the artery at the aneurysm in 21 patients and stent-assisted coil placement in two cases. Parent artery sacrifice was successful in all cases. No patient sustained permanent complications as a result of treatment. Two (8.6%) patients died due to the severity of their original SAH. Their findings were normal in 14 (60.8%) patients (including five of the 12 presenting with poor-grade SAH) at the final follow-up. They concluded that intracranial VA dissecting aneurysms can be managed safely with coil occlusion of the lesion and/or parent artery even in patients presenting with poor neurological condition [25]. In accordance with these results, we treated 12 cases with a VA dissection and only one case died due to a reason unrelated to the procedure. None of the patients had sustained complications, and an improved condition occurred in 100% of the cases at the final follow-up. The endovascular stent-based method achieved stabilization of the dissected artery without sacrificing the artery. This method of extracranial and intracranial carotid artery dissection was supported in a recent study by Ohta et al. [26]. They showed that this procedure is safe and effective, particularly in patients with a symptomatic dissection that is not responsive to medical therapy. Adolescent patients with intracranial ICA dissections do well clinically after stent placement and show no evidence of restenosis on follow-up angiography [27, 28]. In the present study, we treated five cases with ICA dissections that were followed up for 16 months and had good final clinical results (mRS: 0-1, final follow-up angiography: class A) which paralleled the results described previously. Ten patients with spontaneous dissections (nine with VA lesions and one with an ICA lesion) underwent an endovascular approach and stent placement to evaluate treating spontaneous arterial dissections with stent placement for preserving the parent artery. The overall technical success rate was 90%, and no postprocedural complications were observed. These clinical and angiographic follow-up results suggest that stent placement offers a viable alternative to complex surgical or deconstructive procedures [28]. 

We evaluated the endovascular results of our cases after a mean follow-up time of 17 months. In a retrospective analysis of 14 patients with intracranial dissection treated with endovascular procedures with a mean follow-up duration of 21 months, Bourcier et al. concluded that endovascular treatment approaches can be used safely to ensure long-term stability and change the poor prognosis specified by each location [29]. An investigation of treatment options (mainly endovascular treatment and conservative therapy) for hemorrhagic intracranial dissections of 27 patients was conducted during a 16-year period. Occlusion was performed using coils in six dissections, with proximal balloon occlusion in six dissections, and 16 dissections were conservatively managed. No rebleeding occurred after endovascular surgery. Of the 10 patients treated conservatively, four died. They concluded that an endovascular approach provides effective protection against rebleeding but suggested that PAO with balloons and stents should be considered to preserve vessel permeability in specific cases and that occlusion with coils at the dissection site is the current method of choice [30]. 

Some studies have compared surgical treatment or conservative treatment with an endovascular approach. Yonekawa et al. emphasized the significance of aneurysm entrapment combined with bypass surgery for hemorrhagic cerebral dissecting aneurysms; however, they stated that the less invasive endovascular technique is evolving and that its availability and superiority make it an attractive option [31]. Uhl et al. treated 13 intracranial dissecting aneurysms and evaluated management considerations of surgical and endovascular approaches to preserve arterial continuity. The VA was affected in six patients, the BA in two, the ICA in three, the MCA in one, and the A2 segment of the ACA in one. They concluded that conservatively managed patients have a poor prognosis [32]. Araki et al. reviewed 17 case reports of ACA dissected aneurysms. Most ischemic cases had a good prognosis, even when treated conservatively, whereas the hemorrhagic types, who were treated conservatively, had a very poor prognosis [33]. There are also different reports with relatively same results [34, 35]. 

In the current investigation, five patients presented with cerebral ischemia and 25 had SAH. The stent-only technique was applied for 16% of the patients who presented with SAH and for all intracranial dissection cases that presented with ischemia or the mass effect. Procedural-related complications were observed in only two patients. Only 19% had procedural-related complications (distal branch emboli of the PICA and putamen emboli), but their final mRS score and follow-up angiography results were favorable with low mortality rate (3.3%). 

Our series suggests that favorable outcomes can be achieved following applying PAO with coil embolization or stent implants as an endovascular approach for intracranial symptomatic ruptured or unruptured dissecting aneurysms. However, the long-term efficacy and durability of these procedures for arterial dissections remain to be determined in a larger series. 

*The limitations of the current study *included the small number of patients, a lack of comparative data for other treatment options in the control group, the lack of long-term follow-up, and incomplete follow-up data. A randomized, multicenter, parallel trial may be required to assess the clinical efficacy and safety of these procedures for intracranial artery dissection. 

## 5. Conclusion 

We observed favorable outcomes following an endovascular approach for symptomatic ruptured or unruptured dissecting aneurysms. Our series results suggest that PAO with coil embolization or stenting techniques can be recommended for treating intracranial dissections. However, the long-term efficacy and durability of these procedures for arterial dissections remain to be determined in a larger series. 

## Figures and Tables

**Table 1:** Clinical characteristics, angiographic, clinical, and follow-up results of the patients.

No.	Age/sex	Presentation/H&H Score	Vascular RF	Baseline mRS/NIHSS	Location/segment	Treatment	PA-NIHSS	Final MRS/FU (months)	Procedure-related complications	Final F/U Angiography and DSA study
1	56/M	SAH/4	HTN, smoke, HLP	4/15	Lt VA/V4	PAO + CE	14	6/10 days	None	N/A
2	44/F	SAH/4	HTN	3/14	Lt VA/V4	PAO + CE	9	0/24	Distal branch PICA emboli	Class A
3	40/M	SAH/4	Smoke	4/17	Lt VA/V4	PAO + CE	15	0/27	Distal branch PICA emboli	Class A
4	40/M	SAH/3	HTN, smoke	3/9	Rt VA/V4	PAO + CE	7	0/28	None	Class A
5	33/F	SAH/3	None	2/8	Rt VA/V4	PAO + CE	4	0/33	None	Class A
6	50/M	SAH/3	None	3/11	Lt VA/V4	PAO + CE	7	1/32	None	Class A
7	40/M	SAH/3	Smoke, DM	3/12	Lt VA/V4	PAO + CE	6	0/30	None	Class A
8	69/F	SAH/4	HTN, DM, HLP, smoking	4/16	Lt VA/V4	PAO + CE	10	1/19	Distal branch PICA emboli	Class A
9	37/M	SAH/3	HTN	3/10	Lt VA/V4	PAO + CE	7	0/17	None	Class A
10	25/F	Lt cerebellum infarction	None	3/13	Lt VA/V4	S	7	0/19	None	Class A
11	41/F	SAH/2	HTN, HLP	2/6	Rt VA/V4	PAO + CE	3	0/12	None	Class A
12	56/F	SAH/4	HTN, DM	4/15	Lt PCA/P1	PAO + CE	9	1/10	None	Class A
13	46/F	SAH/3	None	3/12	Lt PCA/P1	PAO + CE	7	1/14	None	Class A
14	61/M	SAH/3	HTN, DM	3/13	Rt PCA/P1	PAO + CE	8	1/16	None	Class A
15	60/M	III Nerve palsy	HTN, smoke	1/6	Lt PCA/P1	S	3	0/15	None	Class A
16	37/M	Lt cerebellum infarction	DM, smoke	3/10	Lt VA/V4	S	4	0/13	None	Class A
17	45/F	SAH/3	HTN, DM	3/9	Rt ICA/supracavernous	PAO + CE	4	0/11	None	Class A
18	77/F	SAH/3	HTN, DM	3/11	Rt ICA/supracavernous	PAO + CE	8	1/18	None	Class A
19	64/M	SAH/2	HTN, HLP, smoke	3/7	Lt ICA/supracavernous	PAO + CE	3	0/12	None	Class A
20	73/M	SAH/3	HTN	3/12	Lt ICA/supracavernous	PAO + CE	7	1/24	None	Class A
21	62/M	Ophthalmoplegia	Smoke, DM, IHD	3/9	Rt ICA/supracavernous	S	4	0/17	None	Class A
22	57/M	SAH/3	HTN, smoke	3/9	Rt MCA/M1	PAO + CE	6	0/11	None	Class A
23	55/F	SAH/3	HTN	3/11	Rt MCA/M1	S	5	0/12	None	Class A
24	64/F	SAH/4	HTN, HLP	4/14	Lt MCA/M1	S	9	3/14	Putamen emboli	Class A
25	49/M	SAH/4	HTN, DM	4/17	Lt MCA/M1	S	10	1/13	Putamen emboli	Class C
26	43/M	SAH/3	HTN, DM	3/13	Lt MCA/M2	PAO + CE	9	0/19	None	Class A
27	40/f	SAH/3	HTN, DM	3/9	Rt ACA/A2	S	5	0/13	None	Class A
28	49/F	Dysarthria	HTN, DM	3/13	Lt ACA/A2	S	9	0/8	None	Class A
29	37/F	SAH/3	HTN, DM	4/10	Lt ACA/A2	PAO + CE	5	0/6	None	Class A
30	60/M	SAH/4	HTN, DM	4/15	Lt PCA/P1	PAO + CE	11	1/6	Thalamus emboli	Class A

H&H: Hunt and Hess, SAH: subarachnoid hemorrhage, HTN: hypertension, DM: diabetes mellitus, HLP: hyperlipidemia, mRS: modified Rankin Score, NIHSS: National Institutes of Health Stroke Scale, Lt: left, Rt: right, VA: vertebral artery, PCA: posterior cerebellar artery, ICA: internal carotid artery, MCA: middle cerebral artery, ACA: anterior cerebral artery, PICA: posterior inferior cerebral artery, PA: postangioplasty, PAO: parent artery occlusion, CE: coil embolization, S: stent, FU: follow-up, Class A: complete obliteration (if the dissection sac was completely obviated or the parent vessel completely embolized), Class C: stable (if the dissection sac revealed no progressive remarkable change in size and shape).

**Table 2:** Follow-up angiographic results according to presentation and endovascular therapeutic procedure.

Variables	Final follow-up angiographic results (%)		*P* value
	Improved	Non-improved	
Ruptured-dissection	24 (96)	1 (4)	0.833
Unruptured-dissection	5 (100)	0	
Stent-only Treated	8 (88.9)	1 (11.1)	0.300
PAO with coil embolization	21 (100)	0	
